# Major trends in mobility technology research and development: Overview of the results of the NSF-WTEC European study

**DOI:** 10.1186/1743-0003-9-22

**Published:** 2012-04-20

**Authors:** David J Reinkensmeyer, Paolo Bonato, Michael L Boninger, Leighton Chan, Rachel E Cowan, Benjamin J Fregly, Mary M Rodgers

**Affiliations:** 1 Department of Mechanical & Aerospace Engineering Department of Anatomy and Neurobiology Department of Biomedical Engineering University of California 4200 Engineering Gateway Irvine, CA 92697-3875 USA; 2 Department of PM&R - Harvard Medical School Spaulding Rehabilitation Hospital 125 Nashua Street Boston, MA 02114; 3 Department of Physical Medicine & Rehabilitation University of Pittsburgh School of Medicine 3471 5th Ave, Suite 201 Pittsburgh, PA 15260; 4 VA Pittsburgh Healthcare System 6425 Penn Avenue, Suite 400 Pittsburgh, PA 15206; 5 Rehabilitation Medicine Department NIH Clinical Center 10 Center Drive Bethesda, MD 20892; 6 Department of Neurological Surgery The Miami Project to Cure Paralysis University of Miami Miller School of Medicine 1095 NW 14th Terrace Miami, FL 33136 USA; 7 Department of Mechanical & Aerospace Engineering 231 MAE-A Building, P.O. Box 116250 University of Florida Gainesville, FL 32611; 8 Department of Physical Therapy and Rehabilitation Science University of Maryland 100 Penn Street Baltimore, MD 21201; 9 National Institute of Biomedical Imaging and Bioengineering/NIH 6707 Democracy Blvd. Bethesda, MD 20892-5477

## Abstract

Mobility technologies, including wheelchairs, prostheses, joint replacements, assistive devices, and therapeutic exercise equipment help millions of people participate in desired life activities. Yet, these technologies are not yet fully transformative because many desired activities cannot be pursued or are difficult to pursue for the millions of individuals with mobility related impairments. This WTEC study, initiated and funded by the National Science Foundation, was designed to gather information on European innovations and trends in technology that might lead to greater mobility for a wider range of people. What might these transformative technologies be and how might they arise? Based on visits to leading mobility technology research labs in western Europe, the WTEC panel identified eight major trends in mobility technology research. This commentary summarizes these trends, which are then described in detail in companion papers appearing in this special issue.

## Introduction

Mobility technology plays a critical role in millions of people's lives: consider the impact of a wheelchair on an individual who cannot walk, or of a prosthetic leg on a person with an above- knee amputation, or of a hip replacement on a person who has become sedentary because of the pain associated with joint degeneration. In each case, the technology transforms the person's life because it allows him or her to participate much more fully in desired life activities. Yet, even state-of-the-art mobility technology is not yet fully transformative. As illustrated in the companion paper by Boninger and Cowan, there are still routine activities that cannot be pursued, or are difficult to pursue, by individuals who use wheelchairs, prostheses, or joint implants. In addition, there are people with other physical disabilities, including those caused by age-related impairments, for which the enablement provided by technology is still too limited. This study is about identifying status and trends in technology that will lead to a fuller restoration of movement ability for a wider range of people. What might these transformative technologies be and how might they arise?

The National Science Foundation, working with the World Technology Evaluation Center and the Department of Veterans Affairs, selected a panel of American experts in mobility technology to help answer this question. The assembled team included engineers and clinicians with expertise in a broad range of mobility technologies and included an engineer and a scientist with a physical disability. The team worked with WTEC to arrange a brief (5 days) but intense (33 site visits split between two groups) tour of leading laboratories in mobility technology in western Europe. Western Europe was chosen because of its rich and broad research activity in mobility technology. Note that the panel did not focus on brain machine interface technologies, as NSF had recently sponsored a similar study focused on that technology.

The premise of this methodology was that visiting cutting-edge research sites in Europe in person in rapid succession would afford the team an opportunity to identify trends in mobility technology research while also allowing us to think outside the box of what is currently being done in the United States in this field. We are deeply appreciative of the hospitality, openness, and thoughtful input from our European hosts, which made this study possible.

We first characterized the trends we observed during the October 18-22, 2010 European tour at a workshop held at NSF on November 16, 2010 (video available at http://www.wtec.org). This commentary briefly summarizes the major trends we observed, while the companion reports published in this special issue provide greater detail.

## Major Trends in Mobility Technology Research

### The panel identified eight major trends in mobility technology research

#### 1. Assistive technologies are being designed to integrate more closely with the user, decreasing user burden while increasing user capability

Although the panel saw no fundamentally new assistive technologies, there was much innovation aimed at making existing assistive technologies, including powered wheelchairs, prostheses, functional electrical stimulation systems, and exoskeletons, more seamlessly integrate with the capabilities of the user. While each solution we observed was uniquely conceived for a specific application, in general, seamless integration was often being facilitated by smaller, better movement sensors and embedded computation that took advantage of this sensor data, along with a careful consideration of the fundamental physiology, mechanics, available capacities, and needs of the user. Examples are a prosthetic hand that incorporates a small camera and automatically shapes itself to the object being grasped, a wheelchair that seamlessly shares control with the user, and a functional electrical stimulation system that identifies and cancels tremor in real-time. Better integration decreases user burden while also expanding user capability and, as discussed by Cowan and others in the companion paper, thereby provides a possible route to transformative impact by assisting in the desired life activities of the user.

#### 2. Research on technologies for rehabilitation therapy is growing rapidly and beginning to transform clinical practice. At the same time, the need for therapy technology that can be used at home is largely unmet

Research into new therapeutic technologies, including robotics, virtual reality, and motion-based gaming, has risen rapidly over the past twenty years and continues to evolve rapidly. Such technologies have proven effective at reducing physical impairment, and they make rehabilitation exercise more engaging and less labor intensive. Most sites we visited had major efforts aimed at developing improved therapeutic technologies, and we visited an impressive new rehabilitation hospital in Berlin that was designed explicitly to integrate these technologies closely into clinical practice. Thus, technologies for rehabilitation therapy are a large, if not the largest, thrust area currently in mobility technology research. Nevertheless, these technologies as yet have only limited therapeutic benefit, and the technological transformation of at-home therapy is yet to happen.

As discussed in the companion paper by Reinkensmeyer and Boninger, next generation approaches to improving therapeutic technology include designing technology for early application after injury, designing lower cost devices, developing technology with more degrees of freedom to allow training of more naturalistic movements, and improving control and feedback to enhance movement recovery and motor learning. Wearable systems are being developed to be used while performing daily activities, thereby blurring the traditional distinction between assistive and therapeutic technologies. In the future, people will use assistive technologies both to perform activities of daily living and to assist in recovery, transforming the nature of rehabilitation therapy and meeting the need for therapy outside of the clinic at the same time.

#### 3. There is a fundamental need in mobility technology research for better neuromusculoskeletal models that can be personalized to predict on a case-by-case basis optimal treatments for individuals

As discussed in the companion paper by Fregly and others, current clinical treatment plans are often generic rather than customized to each patient, and this situation likely limits the effectiveness of these plans. The panel observed a significant amount of work aimed at developing personalized neuromusculoskeletal models that can predict outcomes of different treatments, or the effect of different parameters within a treatment, on a patient-by-patient basis. These models are based on the premise that each patient possesses unique anatomical, neurological, and functional characteristics that significantly impact his or her optimal treatment.

There is a particularly large gap in the development of verified, customizable models of neural control, learning, and plasticity; that is, more progress has been made in personalizing muscle and skeletal models, although these too still need development. Thus, the design of many mobility technologies is still largely based on trial-and-error clinical testing because there is a lack of fundamental scientific insight into how different technologies will interact with the human movement control system. Engineers worldwide recognize the immense impact that a detailed, customizable, computational model of human movement control and use-dependent neural recovery, for example, could exert over the design process for mobility medical interventions and technology.

#### 4. Wearable sensors and pervasive systems will improve health and wellness monitoring, safety monitoring, home rehabilitation, assessment of treatment efficacy, and early detection of disorders for people with mobility impairment

Improvements in health care are resulting in increased survival rates from acute trauma as well as in people living longer but with more complex health conditions, including the large population of baby boomers. Thus, many societies have a need to provide complex health care for an increasing number of individuals, many with physical disabilities, and many with reduced access to providers. As discussed in the companion paper by Patel and others, wearable sensors and pervasive sensing systems will improve monitoring of health and safety and will help automate and quantify home rehabilitation. They will also assist in early detection of disorders for people with mobility impairment. Several new technologies are enabling this revolution. Miniaturized inertial sensors are been used in motor activity and other health status monitoring systems, and in smart prostheses and orthoses. Advances in material science have enabled the development of e-textile based systems that integrate sensing capability into garments. Mobile phone technology provides a widespread platform for remote monitoring systems based on wearable sensors. Continued development and integration is needed to provide the usability, safety, reliability, and security needed for home health care.

#### 5. Improvements in actuators and power supplies have not progressed as quickly as those in sensors; the invention of a stronger, lighter, and more efficient actuator and more compact power supply would accelerate assistive and therapeutic technology advances as well as spawn many new applications of mobility technology

A striking trend observed during our visit was that, while new forms of sensors were being routinely applied to improve mobility technology, the actuator technology we observed being used in mobility technology was relatively static. There is a fundamental need for a stronger, lighter, more efficient actuator and a more compact power supply. Mobility at its core is about applying forces, and our current methods for applying forces are relatively bulky and inelegant, and thus limit the environments and situations in which mobility technology can be applied.

#### 6. Eliminating physical impairment will ultimately require combinations of physical training and plasticity/regenerative therapies

There is an increasing recognition that the ability of therapeutic technologies to substantially resolve impairment will ultimately lie in combining these technologies with plasticity-enhancing and regenerative therapies, such as cellular, molecular, or electrical stimulation approaches. As discussed by Reinkensmeyer and Boninger in their companion paper, there is a "science of combination therapies" emerging, which seeks to characterize the complex interactions between training, plasticity, and regeneration. This science will define the conditions under which combined treatments cancel each other, add their effects, or synergize with each other. An important concept that will influence mobility technology design is that the experience of different physical training activities may compete for the new neural resources made available by plasticity or regenerative therapies. In general, future physical therapeutic technologies that are based on the science of combination therapies will stand the best chance of eliminating many forms of physical impairment.

#### 7. Multidisciplinary teams that work closely with consumers and are embedded with scientists with an intimate knowledge of disability are best positioned to produce transformative mobility technology

As observed by Boninger and others in a companion paper, an overwhelming theme that emerged from the trip was that multidisciplinary teams that included, at a minimum, engineers, clinicians, industrial partners, and consumers, were by far the most successful in promoting education, research, and technology transfer in mobility technology. Multiple, multi-disciplinary teams at one site appeared to generate a critical mass, which increased productivity. Experiential learning programs that incorporated the multidisciplinary approach appeared to prepare students best for mobility technology research and development. The panel noted that an important target for funding agencies is to devise strategies for recruiting and training people with mobility impairment to participate in and lead mobility technology research teams. In addition, collaborations across countries, which are required by many European Union funding mechanisms, brought unique expertise and knowledge of different cultures while helping with recruitment. The panel observed that funding agencies have the ability to force change, as exemplified by this presence of multi-country collaboration in Europe, and to assist in commercialization, as exemplified by the tight integration of companies in many of the projects we observed. In fact, as described by Boninger et al in greater detail in the companion paper, the main differences perceived by the panel between U.S. and E.U. work in mobility technology involved just these structural differences: seemingly more frequent involvement of multiple countries and industry in collaborative projects.

#### 8. Finally, government support for research in mobility technology has led to substantial gains. Future and growing support is essential to continued advancement

The vast majority of the research we observed was government funded. This funding has spurred technology that has already transformed the lives of individuals with mobility related impairments. In Europe, there were a number of programs that promote technology transfer and greater ties between industry and researchers. It is clear that continued and increased funding in this growing area of need is essential to continued progress. The support should encourage industry and university collaboration for commercialization of products

## Conclusions

The WTEC panel concludes that research and application of mobility technology will grow dramatically in future years. As evidenced by the trends listed above, researchers in the field have identified and are solving key problems that have limited progress in mobility technology. It is expected that advances in mobility technology will substantially expand participation in desired life activities for a wider range of people in future years.

